# Clinical Efficacy of Therapy with Recombinant Human Interferon α1b in Hand, Foot, and Mouth Disease with Enterovirus 71 Infection

**DOI:** 10.1371/journal.pone.0148907

**Published:** 2016-02-16

**Authors:** Xueyong Huang, Xi Zhang, Fang Wang, Haiyan Wei, Hong Ma, Meili Sui, Jie Lu, Huaili Wang, J. Stephen Dumler, Guangyao Sheng, Bianli Xu

**Affiliations:** 1 Henan Center for Disease Control and Prevention, Zhengzhou, China; 2 Children’s Hospital of Kaifeng City, Zhengzhou, China; 3 Children’s Hospital of Zhengzhou City, Zhengzhou, China; 4 College of Public Health, Zhengzhou University, Zhengzhou, China; 5 The First Affiliated Hospital of Zhengzhou University, Zhengzhou, China; 6 Departments of Pathology and Microbiology & Immunology, University of Maryland School of Medicine, Baltimore, Maryland, United States of America; University of British Columbia, CANADA

## Abstract

A rapid expansion of HFMD with enterovirus 71 infection outbreaks has occurred and caused deaths in recent years in China, but no vaccine or antiviral drug is currently available for EV71 infection. This study aims to provide treatment programs for HFMD patients. We conducted a randomized, double-blind, controlled trial and evaluated clinical efficacy of therapy with rHuIFN-α1b in HFMD patients with EV71 infection. There were statistical differences in outcomes including the fever clearance time, healing time of typical skin or oral mucosa lesions, and EV71 viral load of the HFMD patients among ultrasonic aerosol inhalation group, intramuscular injection group and control group. rHuIFN-α1b therapy reduced the fever clearance time, healing time of typical skin or oral mucosa lesions, and EV71 viral load in children with HFMD.

***Trial Registration*:** Chinese Clinical Trial Registry ChiCTR-TRC-14005153

## Introduction

Hand, foot, and mouth disease (HFMD) is an important infectious disease in young children, particularly in those less than 5 years old. HFMD epidemics have affected various countries in the past 40 years [1]. Large-scale outbreaks have been a serious public health issue in China since 2008 [2]. Human Enterovirus 71 (EV71) and Coxsackievirus A16 (CVA16) are common etiological agents of HFMD in children, but the former can cause severe complications, such as aseptic meningitis, acute flaccid paralysis (AFP), meningoencephalitis and cerebellitis, with mortality rates ranging from 10 to 25.7% [3–5]. A cyclical epidemic of HFMD has been ongoing for 2 to 3 years in the region, EV71 is an important pathogen with increasing health threat to humans [6].

Interferons (IFNs) are natural glycoproteins belonging to the cytokine superfamily, and are produced in response to foreign agents such as viruses, parasites and tumor cells. They are important modulators of the immune response, particularly in inhibiting viral replication within host cells, activating natural killer cells and macrophages, increasing antigen presentation to lymphocytes, and inducing the resistance of host cells to viral infection [7, 8]. Interferon alpha (IFN-α) is a type I interferon; at least 23 variants of IFN-α are known. The individual proteins have molecular masses between 19–26 kDa and consist of proteins with lengths of 156–166 and 172 amino acids. All IFN-α subtypes possess a common conserved sequence between amino acid positions 115–151 while the amino-terminal ends are variable. Recombinant human interferon α1b (rHuIFN-α1b) can be expressed in *E*. *coli* to yield an approximately 19 kDa single non-glycosylated polypeptide chain containing 166 amino acids [9]. rHuIFN-α1b has been used for the treatment of chronic viral hepatitis B, hepatitis C virus (HCV), viral pneumonia and adenovirus infections [10–13].

We undertook a clinical trial in HFMD patients with EV71 infection to examine clinical efficacy of therapy with rHuIFN-α1b. Our studies intended to provide treatment programs for HFMD patients.

## Materials and Methods

### Ethics Statement

The protocol for this clinical trial and supporting CONSORT checklist are available as supporting information; see S1 CONSORT Checklist and S1 Protocol. The study protocol was approved by Chinese Ethics Committee of Registering Clinical Trials (ChiECRCT) on 21 August 2014 and was conducted in accordance with the principles of the Declaration of Helsinki and Good Clinical Practice. The study was registered as a clinical trial at Chinese Clinical Trial Registry (ChiCTR) [Registration number: ChiCTR-TRC-14005153; URL http://www.chictr.org.cn/showproj.aspx?proj=4422]. All the participating children’s parents provided informed consent and signed the informed consent prior to enrolment of their child into the trial, see S1 Consent.

### Recombinant Human Interferon α1b

Recombinant human interferon α1b (rHuIFN-α1b) for injection (Shenzhen Kexing Biotech Co., Ltd, Shenzhen, China) was expressed in *E*. *coli*, purified and processed into a freeze-dried powder including human serum albumin, sodium chloride. The freeze-dried powder is stable when stored and transported at 2–8°C from light. 20 μg of the freeze-dried powder per vial was used for this study (Authorization number: Guo Yao zhun Zi S20033034).

### Patients

#### Sample Size

The incidence of severe cases is about 15% among hospitalized patients with HFMD in Henan province [14]. To base this difference in proportion of responders between control group and experimental groups (nebulization group and injection group), with 80% power and two tailed 5% level of significance, the minimal sample size was calculated to be 72 for each group.

#### Clinical Diagnostic Criteria

HFMD was clinically diagnosed based on the guidelines for control and prevention of HFMD issued by the National Health and Family Planning Commission of the People’s Republic of China (2010). Briefly this includes patients from HFMD endemic regions presenting during the epidemic season; fever; and, skin eruptions on the hands, feet, and mouth, or only in the oral cavity.

#### Inclusion and Exclusion Criteria

Criteria for inclusion were: age between 0.5 to 5 years; according to the clinical diagnostic criteria of HFMD, subject patients with one of following clinical manifestations: 1) fever (axillary temperature ≥ 38.5C); 2) neurological complications including mental fatigue, irritability, headache, vomiting, convulsions, limb weakness; and 3) respiratory frequency (RF) ≥30 under resting conditions, heart rate (HR) ≥ 140; and stool specimen was positive for EV71 by real-time RT-PCR.

Exclusions from participation included: 1) critically ill HFMD patients whose treatment could be complicated by central respiratory failure or who had brain-stem encephalitis, acute flaccid paralysis, neurogenic pulmonary edema, neurogenic shock or other severe symptoms, or required endotracheal intubation and mechanical ventilation; 2) allergic constitution, especially allergy to multiple antibiotics or interferon products; 3) congenital heart disease; and 4) epilepsy and other central nervous system dysfunction.

**Fig 1:**
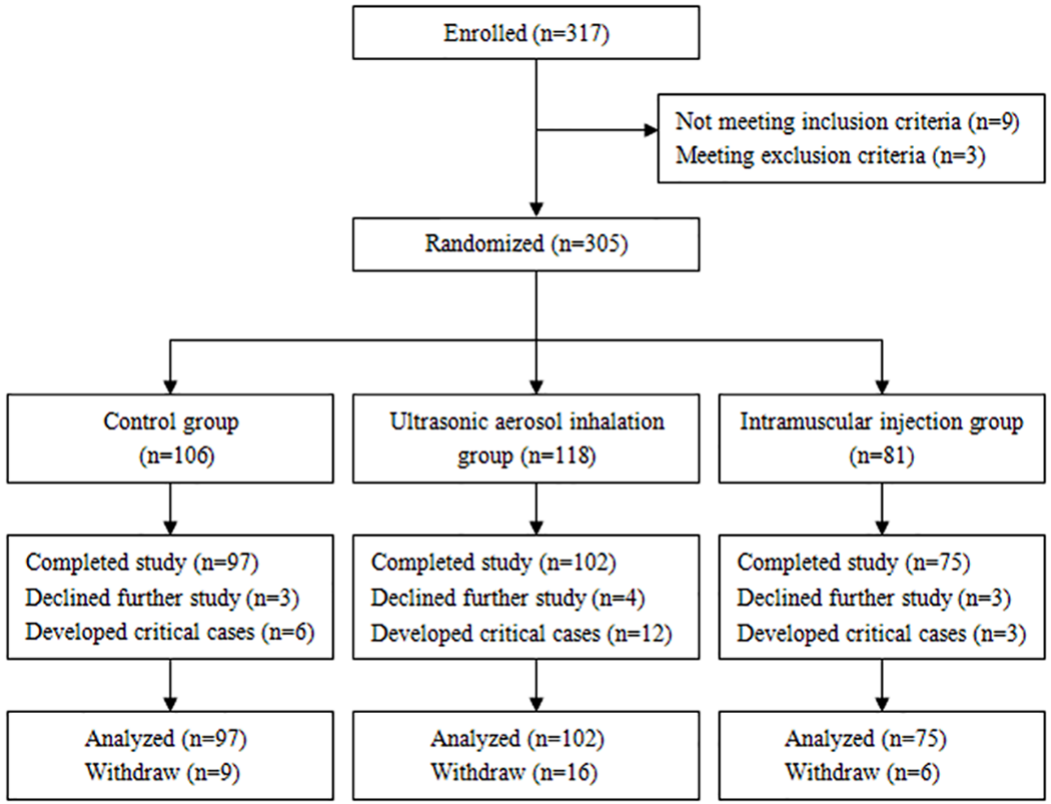
Consort statement flow chart.

Between August 2014 and July 2015, 317 patients with HFMD were registered at Children’s Hospital of Zhengzhou and Children’s Hospital of Kaifeng. The patients were randomized into a control group, an ultrasonic aerosol inhalation group, and an intramuscular injection group. The randomization codes were prepared by the Department of Epidemiology and Statistics, College of Public Health, Zhengzhou University with the use of STATA 8.0 software. The study was conducted in an inpatient setting according to a randomized, double-blind, comparative study design, in which the purpose was to evaluate efficacy of rHuIFN-α1b in patients with EV71 infection (Fig 1).

### Treatment Plan

The patients who met the eligibility criteria were randomized to an ultrasonic aerosol inhalation group, an intramuscular injection group and a control group. All HFMD patients received supportive and symptomatic treatment. Patients randomized to the ultrasonic aerosol inhalation group were administered inhaled ultrasonically-nebulized rHuIFN-α1b (20 μg freeze-dried powder of rHuIFN-α1b dissolved in 4 mL physiological saline) once daily over 15–20 minutes for 5 days. The intramuscular injection group received rHuIFN-α1b (20 μg freeze-dried powder of rHuIFN-α1b dissolved in 1 mL physiological saline) by intramuscular injection for 5 days. The control group only received supportive and symptomatic treatment as per current standard of care. Stool specimens for viral load measurements were collected on days 0 and 6.

### Efficacy and Safety Assessments

The patients were observed from 0 to 5 days and data was recorded in case report forms (CRF) by the investigators, including the following: temperature, skin rashes, oral ulcers, heart rate, respiratory rate, and nervous system signs/symptoms (apathetic, irritability, headache, vomiting, muscular spasms, limb weakness, neck rigidity). During the treatment period, safety was assessed by clinical examination and laboratory investigations, and adverse events were recorded by asking the HFMD subjects’ parents. Adverse events were graded according to the degree of correlation between those events and rHuIFN-α1b.

### Termination Criteria

Termination criteria for the study or for individual patients included: 1) development of critical illness in HFMD subjects; 2) development of intolerable side effects from rHuIFN-α1b in the HFMD subjects; 3) requests from parents for withdrawal from the trial; 4) protocol treatment discontinuation after physician evaluation.

### RNA Extraction and Real-time Quantitative RT-PCR

Total RNA was extracted from clinical samples using the QIAamp viral RNA mini Kit (Qiagen, Germany), following the manufacturer’s instructions. The RNA was eluted in a final volume of 60 μL of elution buffer and used immediately or stored at -80°C.

In order to establish copy number as a unit of viral load, a 226 bp fragment of the VP1 gene of EV71 was amplified by RT-PCR then cloned into vector pGEM-T (Promega, Madison, WI, USA). Next, the recombinant plasmids extracted were linearized by the SpeI digestion (TaKaRa, Dalian, China). The digestion products were both transcribed in vitro into RNAs and purified by a RioMAX large-scale RNA production system-T7 (Promega). Finally, the RNA panels were serially diluted from 2.5 × 10^6^ to 2.5 copies per μL. Since the primers and probe were designed based to amplify the 226 bp cloned fragment, the RNA panels were used as a standard for real-time quantitative RT-PCR and EV71 viral load testing as in our previous studies [15, 16]. The real-time quantitative RT-PCR assay was performed using primers (P1, CCATAAGAACTACACTATGAATATGT, P2, AGTCCACTRCTCACGCTATC), probe (CAGACGTGCACCATCCAGTGAG) and the QIAGEN QuantiTectTM Probe RT-PCR Kit (Qiagen, Germany). The conditions for real-time RT-PCR reaction included 50°C for 30 min, 95°C for 15 min, 45 cycles of 95°C for 20 s, 55°C for 40 s, 68°C for 30 s. Data were analyzed using the software supplied by the manufacturer. The quantitative real-time PCR assay was performed to detect copy number of viral load in stool specimens of the HFMD patients.

### Statistical Analysis

All data was double-entered with the EPIDATA 3.1 software to prevent transcription errors. All statistical analyses were performed using SAS v9.13 (SAS Institute Inc., Cary, NC). Measurement data was analyzed by single factor variance analysis or Student’s t-test. Count data was analyzed by χ^2^ test or Fisher’s exact test. A significant difference was considered with a *P* value less than 0.05.

## Results

### Patient Characteristics

A total of 317 clinically diagnosed HFMD subjects were enrolled in our study; 9 did not meet inclusion criteria and 3 met exclusion criteria. 305 HFMD subjects were then enrolled into the rHuIFN-α1b treatment trial after obtaining written informed consent. The patients were randomized, 106 cases to control group, 118 cases to ultrasonic aerosol inhalation group and 81 cases to intramuscular injection group. 10 HFMD patients withdrew from the treatment trial because of parental request, and 21 HFMD patients withdrew because they developed critical illness requiring transfer to the intensive care unit (ICU). 274 HFMD subjects completed the rHuIFN-α1b treatment trial (Fig 1).

Of the 274 subjects who completed treatment trial patients, 172 (62.77%) were male and 102 (37.23%) were female. The mean age of the HFMD patients was 1.98 years (range, 0.66–5.00). Baseline demographics and clinical characteristics were well matched among the randomized groups (Table 1).

**Table 1:** Demographics and clinical characteristics for participants with HFMD in the randomized groups.

Characteristics	Control group (n = 97)	Ultrasonic aerosol inhalation group (n = 102)	Intramuscular injection group (n = 75)	χ^2^/F Value	*P* Value
**Gender (male/female)**	56/41	66/36	50/25	χ^2^ = 1.704	0.426
**Age (mean ± SD) years**	1.93±0.98	1.89±0.83	2.17±0.74	F = 2.509	0.083
**Weight (mean ± SD) kg**	12.34±2.30	12.20±2.24	12.68±2.66	F = 0.888	0.413
**Temperature (mean ± SD) °C**	38.72±0.64	38.71±0.77	38.68±0.68	F = 0.075	0.928
**Respiratory rate (mean ± SD) times/min**	32.93±3.17	32.10±6.62	32.38±6.67	F = 0.258	0.772
**Heart rate (mean ± SD) times/min**	130.40±11.47	131.55±12.43	128.87±6.97	F = 1.392	0.250

SD: Standard deviation

### Clinical Efficacy

There were statistical differences in outcomes including the fever clearance time, and the healing time for typical skin or oral mucosal lesions. The 3 groups had similar mean scores and no statistical differences for initial body temperature, but the improvement was greater for the ultrasonic aerosol inhalation and intramuscular injection groups compared with the control group at follow up. The mean time to fever clearance was 80.64 hours (95% confidence interval, 76.80 to 84.48) for the control group, 66.00 hours (95% confidence interval, 61.44 to 70.80) for the ultrasonic aerosol inhalation group, and 65.52 hours (95% confidence interval, 60.72 to 70.32) for the intramuscular injection group, respectively. There were statistically significantly differences between the intramuscular injection and the control group (t = 6.275, *P*<0.001), and between the ultrasonic aerosol inhalation and the control groups (t = 6.059, *P*<0.001), but no significant differences between the ultrasonic aerosol inhalation and the intramuscular injection groups (t = 0.216, *P* = 0.873).

The healing time for typical skin or oral mucosal lesions was significantly different between the intramuscular injection group (mean: 61.44 h, 95% CI 57.36 to 65.28 h) and the control group (mean 75.60 h, 95% CI 71.28 to 79.92 h) (t = 5.946, *P*<0.001), and between the ultrasonic aerosol inhalation group (mean 62.88 h, 95% CI, 60.72 to 65.28h) and the control group (t = 5.272, *P*<0.001), but no significant differences were observed between the ultrasonic aerosol inhalation group and the intramuscular injection group (t = 0.675, *P* = 0.536).

The respiratory rates and heart rates were measured every day during clinical trials. The mean respiratory rates were significantly different in the groups on second day, and heart rates were significantly different on third day; there were no significant differences on other days by variance analysis (S1 Table). Generalized estimating equations (GEE) analysis was conducted to examine associations between interact time and groups. The respiratory rates were statistical difference among the groups (Waldχ^2^ = 33.32, *P*<0.001) and interact time (Waldχ^2^ = 202.91, *P*<0.001). When compared by pairwise comparison methods, there was a statistical difference between the control group and other groups or between the ultrasonic aerosol inhalation group and the intramuscular injection group. In interact time, the respiratory rates of first day and second day were not statistical difference, the fourth day and fifth day were also not statistical difference, but the first day and second day were statistically significant difference between the third day, fourth day or fifth day, respectively (*P*<0.05). The mean respiratory rates decreased gradually from first day to fifth day. The heart rates were not statistical difference among the groups (Waldχ^2^ = 2.563, *P* = 0.278) and interact time (Waldχ^2^ = 9.137, *P* = 0.058) by GEE. There were a statistical difference between the first day and third day (*P*<0.05). The mean heart rates increased on second day, but began to decrease from third day (Table 2). No adverse events were observed in 3 groups.

**Table 2:** Generalized estimating equations (GEE) evaluating associations of interact time and groups with respiratory rates and heart rates of the HFMD patient.

Variables	Mean (95% CI)	*P*_*1*_ value	*P*_*2*_ value	*P*_*3*_ value	*P*_*4*_ value
**Respiratory rates (times/min)**					
**Groups**					
Control	29.73 (29.01, 30.46)	Reference			
Ultrasonic aerosol inhalation	28.69 (28012, 29.25)	0.029	Reference		
Intramuscular injection	31.08 (30.49, 31.67)	0.005	<0.001		
**Time**					
First day	32.60 (31.83, 33.36)	Reference			
Second day	32.46 (31.63, 33.30)	0.820	Reference		
Third day	30.08 (29.23, 30.92)	<0.001	<0.001	Reference	
Fourth day	27.52 (26.68, 28.37)	<0.001	<0.001	<0.001	Reference
Fifth day	26.51 (25.65, 27.36)	<0.001	<0.001	<0.001	0.098
**Heart rates (times/min)**					
**Groups**					
Control	129.86 (120.33, 139.38)	Reference			
Ultrasonic aerosol inhalation	126.31 (121.08, 131.55)	0.528	Reference		
Intramuscular injection	132.82 (126.77, 138.87)	0.608	0.111		
**Time**					
First day	129.73 (128.23, 131.22)	Reference			
Second day	140.19 (125.12, 155.26)	0.171	Reference		
Third day	127.18 (125.80, 128.57)	0.014	0.090	Reference	
Fourth day	127.08 (117.17, 137.00)	0.608	0.152	0.985	Reference
Fifth day	124.13 (115.20,133.06)	0.228	0.072	0.513	0.665

### Laboratory Findings

Stool specimens of the HFMD patients were collected on days 0 and 6, and EV71 viral load was detected by the quantitative real-time PCR assay. The mean EV71 viral load was not significantly different at day 0 among groups (F = 439, *P* = 0.645), but was significantly different at day 6 (F = 7.863, *P*<0.001). The mean viral load decrease (the viral load from the second time measurement minus the viral load from the first time measurement) were significantly different among groups (F = 10.076, *P*<0.001), but not significantly different between the ultrasonic aerosol inhalation and the intramuscular injection group (t = 0.8646, *P* = 0.122) (Table 3).

**Table 3:** The results of EV71 viral load testing in Stool specimens of the HFMD patients.

Groups	Day 0 (log10 copies/mL)	Day 6 (log10 copies/mL)	Viral load decrease (log10 copies/mL)
**Control group (n = 97)**	4.76±3.60	3.82±3.19	0.94±3.48
**Ultrasonic aerosol inhalation group (n = 102)**	5.21±4.14	2.67±3.10	2.53±3.43
**Intramuscular injection group (n = 75)**	5.27±4.19	1.87±3.46	3.39±4.17
**F value**	0.439	7.863	10.076
***P* value**	0.645	<0.001	<0.001

## Discussion

EV71 is a small, non-enveloped, positive-stranded RNA virus with a genome of approximately 7,400 bases, and it is a member of the genus Enterovirus in the family Picornaviridae [1]. Since its first isolation in the United States in 1969, EV71 has been identified worldwide as a common cause of HFMD in young children and infants [6]. Large EV71-associated HFMD outbreaks have been reported in the United States, Europe, Australia, and Asia [17]. In China, a large-scale HFMD outbreak gave rise to 2,203,597 reported cases that caused 559 deaths in 2012. Currently, there is neither an approved vaccine nor any effective antiviral drugs for EV71 infection [18].

This randomized, double-blind, controlled trial demonstrates that rHuIFN-α1b combined with supportive and symptomatic therapy greatly decreases viral load, significantly accelerates fever clearance time and the healing time for shin or oral mucosal lesions. No deaths or severe adverse events were observed in rHuIFN-α1b therapy group. Some previous studies showed that ribavirin, several kinds of herbal injections (such as Reduning, Yanhuning, Tanreqing and Qingkailing, etc.) could reduce the time of healing, time of fever clearance, and time for rash to subside. However, the evidence supporting the use of herbal medicine for HMFD is of insufficient quality, and the available data indicate that herbal medicine, especially herbal injections or combinations of herbal and Western medications might improve symptoms of HMFD [19].

When used in the systemic therapy, IFNs are mostly administered by an intramuscular injection. The most frequent adverse effects are flu-like symptoms, especially increased body temperature. EV71 infection causes HFMD, a common exanthema of young children that is characterized by a fever, rashes on the palms and the bottoms of the feet, and ulcers in the oral cavity [20]. As a result of fever as an enrollment criterion, increased body temperature that could have potentially resulted from rHuIFN-α1b treatment could not separately discern in this study.

We observed that the mean respiratory rates and heart rates were significantly different in the groups on the second and third days, respectively. The mean respiratory rates for the intramuscular injection group was higher than for other groups on day 2, and the mean heart rates of intramuscular injection group was higher than that of other groups on third day. This phenomenon could be considered a potential adverse effect of intramuscular rHuIFN-α1b.

There were a number of limitations related to this study, including: lack of long-term follow up after the treatment; and outcome measures during rHuIFN-α1b treatment were only collected for HFMD patients with EV71 infection. Thus, it is premature to conclude that treatment with rHuIFN-α1b for HFMD is safe owing to the lack of long-term follow up. Furthermore, we only investigated hospitalized patients, and these results might not be applicable to the primary care setting.

## Supporting Information

S1 CONSORT Checklist CONSORT Checklist. (PDF)

S1 Consent Informed consent form. (PDF)

S1 Protocol Original study protocol. (PDF)

S1 Table Supporting table. (PDF)
