# Supplementary material for: Clinical Efficacy of Therapy with Recombinant Human Interferon α1b in Hand, Foot, and Mouth Disease with Enterovirus 71 Infection
Source: PLoS One. 2016 Feb 16;11(2):e0148907. doi: 10.1371/journal.pone.0148907 (PMC4755579; doi:10.1371/journal.pone.0148907)
Supplement: S1 Consent — (PDF) [file pone.0148907.s002.pdf]

Informed Consent Form

Dear Parents:

We are doing research on hand foot mouth disease (HFMD), which is very common in our country.

I am going to give you information and invite you to have your child participate in this research. You do not need to decide today whether your child participates or not in the research. Before you decide, you can talk to anyone you feel comfortable with.

Please ask me to stop as we go through the information and I will explain to you the words that you do not understand. If you have questions later, you can ask them for me, the doctors or the staff.

HFMD is caused by a variety of human enteroviruses, it is a deadly infectious diseases and mainly affects oral mucosa and hand and foot skin, and it is classified as class C infectious diseases according to National Health Institutes. Mild HFMD cases are usually self-limiting within a week, with a small number of HFMD complicated by myocarditis, pulmonary edema, heart failure and central nervous system symptoms such as encephalitis, meningitis concurrently, which may leave clinical sequelae such as attention deficit hyperactivity disorder (ADHD) and mental retardation (MR).

At present, symptomatic and supportive treatment is the only clinical treatment for HFMD children, lacking an antiviral treatment that is clinically proven.

Interferon-α is a broad-spectrum anti-virus drug and an immune enhancer approved by SFDA; many domestic hospitals have take interferon-α-1b as conventional antiviral drugs for clinical HFMD patients. The large-scale clinical trials are to demonstrate the efficiency and safety of interferon-α-1b on HFMD, aiming to provide medical evidences for HFMD standardized treatment scheme in our country.

If your child meets our requirements, with your written consent, he(she) may be allocated into the intramuscular injection group, the atomization inhalation group or the control group. In addition, your child will receive symptomatic and supportive treatment as well. Active cooperation is needed for you to report relevant information to the doctor during the observation time.

If you discover your child is with symptoms as follows, please contact doctors related to this research immediately and undergo treatments: (1) continuous high fever (39 ℃, lasting more than 3 days); (2) mental fatigue, vomiting, easily frightened, limb-shaking, limb weakness, standing or sitting instability. (3) tachypnea, breathing slower or abnormalities of respiratory rhythm, with a breathing rate over 30 to 40 times per minute in a quiet condition; (4) cold sweat, cold limbs, skin pattern, increased heart rate(> 140-150 times/min), increased blood pressure; (5) increased peripheral blood WBC count: > 15×10^9/L, and exclusion of infection factors.

We commit that whether the side effect your child may occur is related to our drug or not, he(she) can get timely and effective treatment during the observation. The physician is responsible for distinguishing the expenses during the observation, in which costs related to the drug will be provided free and charged by the bidders and costs on treatments or examinations which are irrelevant to the research will be paid by parents themselves according to the hospital standard.

The free items which are undertaken by the bidders: (1) the costs on side effects related to the drug; (2) all costs including the rescue cost, the treatment and examination cost produced by confirmed serious adverse event (SAE).

Your child's privacy will be protected in accordance with the law, name abbreviation and enrollment NO. rather than his (her) name and identity will appear when used.

You take part in the trail voluntarily, and you can quit at any time. Your decision has no adverse effect on you and your child, that is to say, your child can continue to

receive other treatments.

(Ⅰ) Consulting

If you have any comment to this research, including the drug adverse reactions and other questions on this research, you can contact physician at any time.

Doctor ____________ Telephone_____________.

(Ⅱ) Risks and Benefits

This product has been approved by FDA for antiviral therapy since 10 years ago, and its safety has been confirmed, so there is little risk for your child's participation in the trial, and the outcome of your child will contribute to the effective treatment of other children with HFMD.

Signature of Consent

As the guardian of the child, I have read the foregoing information before signing. I voluntarily take part in the trial and I will cooperate with the researchers widely.

Signature of Guardian ________________ Date ______________ Day/month/year

Signature of Researcher _______________ Date ______________ Day/month/year

Telephone of Researcher_______________(Office) _______________(Mobile phone)
