# Supplementary material for: Clinical Efficacy of Therapy with Recombinant Human Interferon α1b in Hand, Foot, and Mouth Disease with Enterovirus 71 Infection
Source: PLoS One. 2016 Feb 16;11(2):e0148907. doi: 10.1371/journal.pone.0148907 (PMC4755579; doi:10.1371/journal.pone.0148907)
Supplement: S1 Protocol — (PDF) [file pone.0148907.s003.pdf]

Clinical curative effects of recombinant human interferon α-1b on enterovirus 71 infection in children with hand, foot and mouth disease

Experimental Scheme

Critical clinical collaboration of HFMD prevention, Henan province

The lead experts of the subject: the First Affiliated Hospital of Zhengzhou University

The unit participated in the study: 1. Children's Hospital of Kaifeng City
2. Children's Hospital of Zhengzhou City
3. Institute for Infectious Diseases, Henan Center for Disease Control and Prevention

Experimental design and Data statistics: Department of Epidemiology and Biostatistics, College of Public Health, Zhengzhou University

2014.4

ABSTRACT

Trial topic: Clinical curative effects of recombinant human interferon α-1b on enterovirus 71 infection in children with hand, foot and mouth disease.

Generic Name of Experimental Drug: Recombinant human interferon α-1b

Trial purpose

To assess the efficacy and safety of recombinant human interferon α-1b on enterovirus 71 infection in children with hand, foot and mouth disease (HFMD).

Trial design

A prospectively stratified, randomized, double-blind, controlled, clinical trail with blank control was conducted.

Subjects

Children clinically diagnosed with HFMD who were hospitalized for treatment (0.5-5 years).

Sample size

The number to be enrolled is expected to be 360, there were 120 cases each according to the allocation principles 1 : 1 : 1.

Therapeutic schedule

Ultrasonic aerosol inhalation group: Patients randomized to the ultrasonic aerosol inhalation group were administered symptomatic and supportive treatment and inhaled ultrasonically-nebulized rHuIFN-α1b (20 μg freeze-dried powder of rHuIFN-α1b dissolved in 4 mL physiological saline) once daily over 15-20 minutes for 5 days. Compressed-nebulized-inhalation of rHuIFN-α1b, 20μg a time, once a day for five consecutive days of treatment.

Intramuscular injection group: The intramuscular injection group received symptomatic and supportive treatment as well as rHuIFN-α1b (20 μg freeze-dried powder of rHuIFN-α1b dissolved in 1 mL physiological saline) by intramuscular injection, once a day, five consecutive days of treatment.

Control group: Symptomatic and supportive treatment.

Treatment period: five days.

Symptomatic supportive treatment: control body fluid intake, for patients with intracranial hypertension, dehydration diuretics such as mannitol are given, and for patients with pulmonary edema by X-ray, furosemide is given. The children with axillary temperature over 38.5 ℃ are given acetaminophen antipyretics. Gamma globulin and ribavirin (10mg freeze-dried powder of ribavirin per kg body weight per day dissolved in 100 mL physiological saline by intravenous injection in 2 divided doses) should be provided to the patient who behaves spinal cord or rapid processing. No anti-viral traditional Chinese medicine is allowed during the observation period.

Index of curative effect assessment

(1) Fever clearance time; (2) Herpes healing time; (3) Changes of EV71 viral load.

Safety assessment

To observe the changes of laboratory indexes between the pre- and post-therapy including the blood routine and the hepatic function panel which is composed of a group of seven tests(serum alanine aminotransferase (ALT), total protein (TP), albumin (ALB), globulin (GLB), total bilirubin (TBIL), albumin-globulin ratio(A/G), alkaline phosphatase (AKP)). During the period of observation, doctors keep daily inquiries and records of the adverse events of the subjects and evaluate the possible correlation between the adverse events and the drug studied and drug combination. The adverse events are graded into 5 levels: definitely related, possibly related, possibly unrelated, irrelevant, unable to judge.

Statistical analysis:

All data will be double-entered with EpiData 3.0 software to prevent transcription. All statistical analysis are performed on SPSS 15.2. To analyze the general information, human demography, other baseline data, efficacy and safety. Measurement data is analyzed by student-t test and chi- square test or Fish exact test is provided to analyze count data.

Expected schedule:

It might take 9 months to carry out the case observations and treatment records, and the data entry and analysis will be subsequently performed and finished within 6 months, and then to write summary report, indicating that the project may be well done within 15 months from the beginning to the end.

1 Background

Hand foot and mouth disease (HFMD) is a viral disease which is characterized by rashes of hands, feet, and/or herps of oral mucosa. It is mainly caused by enteroviruses, among which Coxackievirus A16 (CA16) and enterovirus 71 (EV71) are the most common. The disease was first reported in the United States in 1969, it wasn't until 1986 that HFMD was confirmed in China. This disease happens all the year round, peaking in late spring or early summer, with strong infectivity and complex transmission, and it is prone to outbreak, HFMD usually occurs in children less than 5 years old, and the prognosis is generally good. But in recent years, HFMD epidemics in the Asia-Pacific region had high case fatality rate [1], among whom the severe cases can be complicated by myocarditis, central nervous system, and multiple organ damage, which can cause death and leave sequela [2]. In 2008, EV71 was the dominant pathogen of the HFMD outbreak in Fuyang City, Anhui Province, epidemiological data showed that there were 9439 HFMD cases with a case fatality rate of 0.24% (23/9439)[3]. In the past three years, the outbreaks of HFMD have been reported in many provinces and regions in China with an increased complications and mortality rate. It is urgent and necessary for clinicians to explore an effective clinical therapy to prevent the occurrence of the severe HFMD cases.

EV71 and CA16 are common etiological agents of HFMD in children, among which the later mainly causes mild HFMD and the former is the dominant pathogen of severe cases. According to the molecular epidemiology on EV71 and molecular genetics of virulence conducted by Solomon in the Asia-Pacific region and beyond in recent 12 years[4], showing that there were 4 subtypes of EV71 (A, B, C and D subtype), the non-structural proteins of different subtypes were associated with the

clinical manifestations of severe HFMD cases, the virus that caused HFMD cases in China mainly belonged to subtype C[5,6], the pathogenesis of the severe manifestations and the EV71 virulence showed that the interaction between host susceptibility and the non-structural protease of virus subtype eventually determined the degree of the patient's clinical condition. A number of basic researches in vitro and in vivo speculated that for severe cases, EV71 mainly went through deep lymphatic tissues in nasopharynx and the intestinal mucosa lamina propria and mesenteric lymphatic tissues, invading to local nerve plexus, and then ascending to the spinal cord, brain stem and cerebral cortex along the axons of nerve plexus, and eventually causing inflammation[4].

At present, there is a lack of evidence-based antiviral drugs and rescue measures for clinical treatment of HFMD, especially for the rescue and treatment of severe cases. Experimental studies have shown that interferon alpha and ribavirin can improve the survival rate of EV71-infected mice and make viral load decreased [7,8], and intravenous immunoglobulin (IVIG) have non-specific anti-inflammatory effects, which helped to improve the condition of severe HMFD cases [9-12]. Although "HFMD diagnosis and treatment guidelines" issued by National Heath and Family Planning Commission of the People's Republic of China in 2010 and 2011 proposed for two consecutive years that traditional Chinese medicine had the effects of heat-clearing, detoxicating and deprive the evil wetness and western medicine also had antiviral effects[13], the recommendation is just on the basis of basic research results and expert consensus.

Interferon(IFN) is the first immune response produced by the host upon infection, and it is a cytokine with multiple biological activities which include anti viral, anti tumor, and immune activities and so on. CHO[14] found that the IFN inducer poly(I:C) can reduce the viral load of newborn mice infected by CoxB3 and prolong the survival time of the infected mice, however, the time of antibody administration in relation to the infection cycle was important, this protective effect was incomplete and could only be demonstrated in animals treated before infection (24h) or shortly after (2h) infection. A study conducted by Liu ML [7] in Taiwan in 2005 reported IFN-α

can significantly increase the survival rate of EV71-infected mice, there was a negative correlation between the serum level of the IFN-α and the viral load, but the protective effect can be antagonized by type 1 IFN-neutralizing antibody. It was supposed that IFN-α played an important role in inhibiting EV71 virus replication and improving the non-specific immunity of infected host.

There were some studies on efficacy and safety of atomization inhalation of interferon a1b, the intramuscular injection and ultrasonic aerosol inhalation conducted by Zhang[17] were used to compare pharmacokinetics, respectively, to observe the drug concentration at 0.5 hpi, 1 hpi, 2 hpi, 3 hpi, 4 hpi, and 8 hpi in lung and plasma, showing that the drug concentration of peripheral blood is equal to intramuscular injection at 3 hpi, and subsequently the former were higher than the later. A tolerance and safety study on effects of atomization inhalation of rHuIFN-α on viral pneumonia conducted by Sun[18] demonstrated that it was safe for pneumonia-infected infants who were administered atomization inhalation once a day with a dosage of 2 μg per kg body weight, or twice a day with a dosage of 1.5 μg per kg body weight, and the vital signs, the laboratory indexes, urine and stool routine results of each group were all in the normal range, no clinical significant changes were observed. Atomization inhalation of IFN-α can achieve an effective plasma concentration against the virus, and can also avoid side effects such as fever, flu-like symptoms caused by the intramuscular injection.

There have been several reports of interferon on HFMD treatment, it was reported by Chen[17] that IFN-α by intramuscular injection for 5 consecutive days (5-100,000U per kg body weight daily) can significantly shorten fever clearance time and herpes healing time, and improve the appetite. It was reported by Huang[18] that the clinical effective rate of fever clearance time and herpes healing time were higher and less cases complicated with central nervous symptoms compared with that of the control group when IFN-α1b was used in the HFMD treatment. However, there was a lack of viral diagnosis and quantifiable objective indicators in the clinical studies, and the curative effect of IFN on severe HFMD cases have not been reported yet.

In our study, EV71-infected HFMD children were collected as the subjects,

aiming to evaluate the efficacy and safety of IFN-α1b on EV71-infected children.

2 Trail purpose

To evaluate the efficacy and safety of rHuIFN-α1b on EV71 infection in children with HFMD.

3 Period and premature termination

It might take 9 months to carry out the case observations and treatment records, and the data entry and analysis were subsequently performed and might be finished within 6 months, and then to write summary report.

The sponsor of the trial reserves the right to terminate the study in advance because of persistent breaches of clinical requirements, improper enrollment or administration or others related to ethics. When it happens, we will take the necessary measures to ensure the interests of subjects after deliberation and consultation by both sides. In this case, all the acquired data is analyzed and the final report is to be completed.

4 Subject recruitment and discontinued cases

4.1 Diagnostic criteria

HFMD diagnosis is as per the HFMD prevention and control guide (2010 edition) issued by National Heath and Family Planning Commission of the People's Republic of China in 2010 [18] and Zhu Futang textbook of pediatrics [19], all cases should meet the following 3 criteria:

(1) Epidemiological data, patients from HFMD endemic regions presenting during the epidemic season;

(2) With or without fever, characterized by low and moderate fever, accidentally high fever;

(3) Skin eruptions on the hands, feet, mouth, and buttock;

4.2 Inclusion Criteria

(1) Age between 0.5 to 5 years old;

(2) According to the clinical diagnostic criteria of HFMD;

1) Fever(axillary temperature≥38.5℃);

2) Neurological complications including mental fatigue, irritability, headache,

vomiting, convulsions, limb weakness;

3) Respiratory frequency (RF) ≥30 under resting conditions, heart rate (HR) ≥ 140;

4) Stool specimen was positive for EV71 by real-time RT-PCR.

4.3 Exclusion Criteria

(1) Critically ill HFMD patients whose treatment could be complicated by central respiratory failure or who had brain-stem encephalitis, acute flaccid paralysis, neurogenic pulmonary edema, neurogenic shock or other severe symptoms, or required endotracheal intubation and mechanical ventilation;

(2) Allergic constitution, especially allergy to multiple antibiotics or interferon products;

(3) Congenital heart disease;

(4) Epilepsy and other central nervous system dysfunction.

4.4 Dropping-out conditions

4.4.1 Discontinued cases

(1) Discontinued cases by researchers

1)A serious drug-related adverse events occur, he(she) should quit according to the doctor's judgment;

2)Some co-morbidity, complications, or special physiological changes take place during the observation, which can affect curative effect and safety judgment;

3)The subjects' compliance is poor, or they themselves change their dressing midway or use traditional Chinese medicine (TCM) that is banned in this trial;

4)Cardiopulmonary failure occurs, who is in need of rescue in intensive care unit(ICU).

(2)Self-quitters

1) Whatever the reason, children's parents propose to the doctor-in-charge that they would like to terminate;

2) The children cannot tolerate the side effects of the medicine.

4.4.2 Disposal of drop-out cases

For discontinued cases, active measures should be taken to accomplish the last test as quickly as possible, in order to analyze the efficacy and safety. The conclusions and reasons for all drop-out cases should be fulfilled in CRF. Generally, there are 6 reasons as follows, adverse events(including drug adverse reaction and allergic reactions), a lack of efficacy(disease worsened or complicated), breach of experimental program(including poor compliance), attrition(including self-quitting on the requirement of their parents), terminated by the sponsor and others.

5 Experimental design

5.1 Experimental design(shown in Annex 1)

A prospectively stratified randomized clinical trail with blank control was conducted.

5.2 Group

(1) Ultrasonic aerosol inhalation group：Patients randomized to the ultrasonic aerosol inhalation group were administered symptomatic and supportive treatment and inhaled ultrasonically-nebulized rHuIFN-α1b (20 μg freeze-dried powder of rHuIFN-α1b dissolved in 4 mL physiological saline) once daily over 15-20 minutes for 5 days. Compressed-nebulized-inhalation of rHuIFN-α1b, 20μg a time, once a day for five consecutive days of treatment.

(2) Intramuscular injection group：The intramuscular injection group received symptomatic and supportive treatment as well as rHuIFN-α1b (20 μg freeze-dried powder of rHuIFN-α1b dissolved in 1 mL physiological saline) by intramuscular injection, once a day for five consecutive days of treatment.

(3) Control group: Symptomatic and supportive treatment.

Period of treatment: five days.

Symptomatic supportive treatment: control body fluid intake, for patients with intracranial hypertension, dehydration diuretics such as mannitol are given, and for patients with pulmonary edema by X-ray, furosemide is given. The children with axillary temperature over 38.5℃ are given acetaminophen antipyretics. Gamma globulin and ribavirin (10mg freeze-dried powder of ribavirin per kg body weight daily dissolved in 100 mL physiological saline by intravenous injection in 2 divided

doses) should be provided to the patient who behaves spinal cord or rapid processing. No anti-viral traditional Chinese medicine is allowed during the period of observation.

5.3 Sample size

According to sample size calculation formulas of non-inferiority tests($n_2 = \frac{(z_{\alpha/2}+z_\beta)^2}{\epsilon^2}\left[\frac{p_1(1-p_1)}{\kappa}+p_2(1-p_2)\right]$, $n_1 = \kappa n_2$), test level α = 0.05 (two-tailed), test power(1-β)=0.8; the sample size of case group and control group is to be allocated according to the allocation principles of 1 : 1 : 1; the clinical cutoff value(△) is -0.05, referring to the clinical experience, the incidence rate of critically ill HFMD cases was circa 15%(supposing that it is the incidence of severe HFMD in the control group). It is supposed that the incidence rate of severe cases can be reduced to 5% after symptomatic and supportive treatment combined with interferon α-1b inhalation therapy, according to the allocation principles 1 : 1 : 1, 72 cases each group, in addition, considering 20% cases of attrition and error filling, the total sample is 264.

5.4 Randomized design

5.4.1 Randomization method

In order to ensure the balance of the experimental group and control group, a completely randomized grouping method will be used in the trail. It is divided into three groups including ultrasonic aerosol inhalation group(A), intramuscular injection group(B) and the control group (C). There are two participated hospitals(Zhengzhou Children's Hospital and Kaifeng Children's Hospital), regarding the imbalance of the two regions(including the regions and therapy on severe HFMD), two random numbers are generated by random number table on SPSS 15.2, respectively, the sequence of each random number is 150.

5.4.2 Randomized assignment and implementation on SPSS

Objective Two random number tables are generated, each random number table containing 150 numbers, 132 cases of each center will be assigned to group A, group B and group C.

Software SPSS

Protocol(Referencing to "SPSS for randomized experiments to design group" in

Chinese Health Statistics, 2005, 22(6))

- SET SEED = **** ****.
- INPUT PROGRAM.
- LOOP INDEX = 1 TO 200
- COMPUTE RANDOM = UNIFORM(200).
- END CASE.
- END LOOP.
- END FILE.
- END INPUT PROGRAM.
- AUTORECODE VARIABLES=RANDOM/IN TO RANK..
- SORT CASES BY RANK(A)..
- RECODE RRANDOM(1 THRU 50=1) (51 THRU 100=2) (1401 THRU 150=3) INTO GROUP.
- EXECUTE.

Results: seen in Annex 2.

5.4.3 Manner of random allocation

Objective: Researchers who are responsible for recruiting and assessing the subject are blind to the allocation order.

Procedure

(1) 150 numbers and the distribution details of each site are generated by the completely random grouping method using SPSS 15.2.

(2) The serial numbers assigned by a random number are put into a sealed opaque envelope in which aluminum foil blocks light through the envelope; the surfaces of envelopes are marked with the distribution number, and the pages of random numbers and the corresponding case or control group are inside.

(3) For each study site, there is a director responsible for the trail who receives the envelopes.

(4) When subjects who meet the standard are recruited, to inform the director of the study site, and encode the subjects according to their temporal orders of enrollment, and then correspond with the random number table to determine the allocation and

inform the executives of the study .

5 Experimental items

5.5.1 Screening/baseline visits(0 day)

(1) Demographic indicators: age, gender, weight.

(2) Primary diseases, drug allergic history(antibiotics, interferon).

(3) Clinical symptoms: fever, mental fatigue, irritability, headaches, vomiting, c onvulsions, limb weakness and so on.

(4)Vital signs of the subjects: temperature, blood pressure, heart rate, respiratory rate and pulse, temperature refers to the highest one among the four axillary temperatures taken in the first hospitalized day, blood pressure, heart rate, respi ratory rate and pulse refer to the counts measured the time when the temperat ure is the highest.

(5) Herpes on the skin or in the oral cavity graded by parts(hands, feet, buttocks, and oral mucosa) and herpes levels(red, pink, dark and fading).

(6) Blood routine, electrocardiogram(ECG), hepatic function panel including a group of seven tests, chest X-ray, peripheral WBC, blood glucose, among which seven tests of hepatic function panel include alanine amino transaminase (ALT), total protein (TP), albumin (ALB), globulin (GLB), total bilirubin (TBIL), albumin/globulin (ALB/GLB), alkaline phosphatase(AKP).

(7) Swab specimens are collected to do qualitative analysis of EV71 within 24h after grouping;

(8) The first group-in stool samples are collected to determine the quantitative analysis of EV71 virus;

(9) The researchers determine whether the children enroll or not, informed consent are written, and then to prescribe the medicines and related drugs from pharmacy department.

5.5.2 Treatment period (1-5 days)

(1) To make records of vital signs(temperature, heart rate, respiratory rate, blood pressure), temperature refers to the highest one among the four axillary temperatures(8:00am, 12:00 am, 16:00pm, 20:00pm) taken in the first hospitalized

day, and blood pressure, heart rate, respiratory rate and pulse refer to the counts measured the time when the temperature is the highest.

(2) The manifestations of nervous system(e.g. mental condition, headache, convul sions, limb weakness).

(3) Skin pattern and cold sweat, and capillary refill time (CRT).

(4) To observe and make records of herpes on the skin or in the oral cavity and graded them by parts(hands, feet, buttocks, and oral mucosa) and herpes levels(red, pink, dark and fading).

(5) Stool specimens of each defecation is collected to do the quantitative analysis of EV71 virus.

(6) Throat swabs are collected every day before ultrasonic aerosol inhalation, and that of control group are collected before 17:00 each day.

(7) To make records of adverse events and correlation analysis was conducted between the drug studied and the adverse events, and record combined medication.

5.5.3 Endpoint of critically ill HFMD cases: enroll the ICU or recover

(1) To observe the children's clinical condition, cases who meet the critically ill criteria will terminate the treatment, and the condition will be assessed by PICS score.

(2) The first stool specimens of group-out children who are critically ill or finish the observation were collected to do the quantitative analysis of EV71 virus and record the time span between the group-out time and the stool collection time(hours).

5.6 Drug withdrawal criteria

(1) Cases who meet the critically ill HFMD criteria.

(2) Cases who fulfill the period of treatment.

(3) Drop-out cases.

6 Evaluation of the curative effect

(1) Fever clearance time

It is the time that axillary temperature of enrolled children returns to normal body temperature (≤37.4 ℃);

(2) Herpes healing time

It refers to the time from rash found when children were enrolled to 80% of rashes

fading;

(3) Changes of EV71 viral load

To compare the changes of EV71 viral load between the first in-group and out-group stool samples of the two groups, and to analyze the correlation between clinical condition and viral load;

6.3 Safety evaluation

To observe the changes of blood routine and the hepatic function panel with a group of seven tests(ALT, TP, ALB, GLO, TB, ALB/GLO, ALP);

During the observation period, doctors keep daily inquiries and records of the adverse events of the subjects and evaluate the possible correlation between the adverse events and the drug studied and drug combination.

6.4 Rescue and treatment of severe / critically ill cases

The severe and critically ill subjects will be treated as per the measures promulgated by "the expert consensus on severe HFMD cases caused by EV71" issued by National Heath and Family Planning Commission of the People's Republic of China in 2011, such as gamma globulin, vascular active drugs, oxygen therapy, mechanical ventilation .

7. The experimental drug

7.1 Drugs of experimental group:

Generic Name

Recombinant human interferon α-1b

Proprietary Name

Sinogen

English name

Recombinant Human Interferon α1b for Injection

Main components

Recombinant human interferon α1b (rHuIFN-α1b) for injection was expressed in *E. coli*, purified and processed into a freeze-dried powder.

Character

White shell -like loose body, once dissolved in 1ml sterile water for injection, it rapidly reconstitutes as clear liquid.

Indications

It has been used in the treatment of viral diseases and certain malignancies. It has been approved for the treatment of chronic hepatitis B, hepatitis C and hairy cell leukemia. The clinical trials and literature have shown that it is effective for the treatment of viral diseases such as herpes zoster, genital warts, epidemic hemorrhagic fever and pediatric respiratory syncytial virus pneumonia and others. It can be also used to treat malignancies such as chronic myeloid leukemia, melanoma, lymphoma.

Specification

one 10μg bottle, one 20μg bottle, one 30μg bottle, one 40μg bottle, one 50μg bottle, one 60μg bottle

Dosage and Administration Each dissolved in 1ml sterile water for injection, intramuscular or subcutaneous injection. Dosage and efficacy are as follows:

Chronic Hepatitis B: 30-50μg/time, every other day, given subcutaneously or intramuscularly with a treatment period of 4-6 months, and the period may be extended to one year according to the disease condition. Available for induction therapy, which means treatment starts with daily 1 bottle and continues 1 bottleevery other day from 0.5-1 months later to the end of treatment period.

Chronic Hepatitis C: 30-50μg/ time, every other day, given subcutaneously or intramuscularly with a treatment period of 4-6 months, the drug will be abandoned when there is no effect for some one. For patients with effective effects, the treatment can be continued for 12 months, and it may be extended to 18 months according to the disease condition. In the first treatment month,1 time/day. After the treatment period, follow-up is done in the next 6-12 months. This treatment should be used to acute hepatitis C earlier, which can reduce chronicity.

Chronic myelogenous leukemia: 30-50μg/time, once a day, given subcutaneously or intramuscularly with a continuous medication for 6 months or more. It can be adjusted according to the disease condition, and changed every other day after remission.

Hairy cell leukemia: 30-50μg/time, once a day, given subcutaneously or

intramuscularly.

Condyloma acuminatum: 10-30μg/ time, given subcutaneously or intramuscularly, or 10μg daily injection under local warts, every other day for 3 consecutive weeks a course. According to the disease condition, the course can be extended or repeated.

Cancer: 30-50μg/time, once a day or every other day, given subcutaneously or intramuscularly with a continuous medication period for six months or more, treatment can be extended as per the disease condition. If the patient's condition doesn’t deteriorate rapidly or no severe adverse reactions occur, continuous treatment should performed at appropriate dosages .

Adverse reactions

Adverse reactions of the product is mild, the most frequently reported adverse reactions are fever and fatigue, which often appear at the initial treatment, mostly one-time and reversible reaction; other possible adverse reactions are headache, muscle pain, joint pain, lack of appetite, nausea and loss of hair, and a few patients may experience blood abnormality such as neutropenia, thrombocytopenia, which can resume when the drug is discontinued, if severe adverse drug reactions(ADR) that the patients can’t tolerate occur, to reduce the dose or discontinue the drug, and the necessary symptomatic treatment is provided.

Contraindications

(1) Cases who are allergic to interferon

(2) Cases who have a history of angina , myocardial infarction and other serious cardiovascular disease

(3) Cases who can not tolerate the side effects of this product because of other serious illnesses

(4) Cases with epilepsy and other central nervous system disorders

Precautions

(1) Cases of allergic constitution, especially those who are allergic to a variety of antibiotics, this product should be used with caution. If severe allergic reactions occur, the drug should be discontinued immediately and appropriate therapy be given.

(2) before use, carefully checking should be done to bottles, if bottles are broken or

cork cracks, it can not be used, after the addition of sterile water for injection, a little shake can make the product dissolve well, if floccule and mass that do not dissolve are seen in the bottle, just abandon it.

(3) Once dissolved, it should be used immediately and graded use is forbidden.

Pharmacokinetics

The peak plasma concentration of healthy volunteers with 60μg injection is 3.99hpi, with an absorption half-life 1.86 hours and a half-life of elimination 4.53 hours. This product is distributed in various organs after absorption. The concentration of injection site is the highest, followed by kidney, spleen, lung, liver, heart, brain and adipose tissue, and it then degrades in vivo, less excretion from urine, stool and bile.

[Storage]: Store and transport at 2-8 ℃ protecting from light.

[Package]: Vials, and 1 each small box

【Validity】 30 months

[Approval Number]: 10μg：Guoyaozhunzi S10960058

20μg：Guoyaozhunzi S20033034

30μg：Guoyaozhunzi S10960059

40μg：Guoyaozhunzi S20033039

50μg：Guoyaozhunzi S10970070

60μg：Guoyaozhunzi S20033035

[Manufacturer] : Shenzhen Kexing Biotech Co., Ltd (Shenzhen, China)

[Drug control group]: Blank

7.3 Drug storage

According to the hospital drug management system, the experimental drug is stored according to the storage condition (2-8 ℃), and the drug is purchased and managed centralized from the pharmacy department in the normal way.

7.4 Drug delivery and number checking

According to medication plan of random number table, researchers receive a daily dose from the pharmacy department every day, the researchers should well

record the number of drugs during the study period, which is used to determine the medication compliance of the subject, it is beneficial to judge the efficacy and safety of the drug. The monitors should conduct an inventory audits to ensure proper use of the study drug (drug apply record sheet in Annex 3).

7.5 Symptomatic and supportive treatment medication

Symptomatic supportive treatment: control body fluid intake, for patients with intracranial hypertension, dehydration diuretics such as mannitol are given, and for patients with pulmonary edema by X-ray, furosemide is given. The children with axillary temperature over 38.5℃ are given acetaminophen antipyretics. Gamma globulin and ribavirin (10mg freeze-dried powder of ribavirin per kg body weight daily dissolved in 100 mL physiological saline by intravenous injection in 2 divided doses) should be provided to the patient who behaves spinal cord or rapid processing. No anti-viral traditional Chinese medicine is allowed during the period of observation.

8 Safety Assessment-records and evaluation of adverse events

In each follow-up, the potential side effects should be monitored and recorded. Adverse events documented during the treatment should be paid more attention to.

8.1 Definition of adverse events

From the time when patients write the informed content and group-in the trial to the end of the administration, any adverse medical incident reported by the patient or observed by the physician should be recorded, whatever there is a causal relationship between the medical incident and the medication, they are regarded as adverse events.

8.2 Methods to acquire adverse events

(1) During the trail, close observation and inquiry should be given to the adverse events of the patient.

(2) Physician-in-chief should report the adverse events they directly observe or the patients report in medical nomenclature.

8.3 Records of adverse events

From the time when patients write the informed content and group-in the trial to the end of the administration, any adverse event (AE) occurring (including clinical adverse events and other adverse events) should truthfully fill in the " Adverse Event Reporting Form" , and record the occurrence time of adverse events (or acquisition time of test sample), the severity, the end time, the relationship between the study drug and adverse events, and the measures that were taken.

8.4 Criteria of adverse events associated with the experimental drug

Five - category classification is used to assess the association between adverse events and experimental drug, including "definitely related, probably related, possibly related, possibly unrelated, irrelevant". The former three are related to the experimental drug, when calculated the incidence of adverse reactions, the three are together as a molecule, all subjects used to evaluate the drug safety as the denominator.

Table 1. Causality criteria of adverse reactions

Standard	definitely related	probably related	possibly related	possibly unrelated	irrelevant
Reasonable time sequence	Yes	Yes	Yes	Yes	No
The type of reaction of drugs is known	Yes	Yes	Yes	No	No
The removing reasons can be improved	Yes	Yes	Yes/No	Yes/No	No
Administration can be repeated again	Yes	?	?	?	No
The reaction may have another explanation	No	No	No	Yes	Yes

8.6 Observation of serious adverse events(SAE)

8.6.1 Determination of SAE

Any event occurs in the time span when patients write the informed content and group-in the trial to the end of the administration, if it meets one or more of the

following criteria, whether it is related to the experimental drug or not, it is defined as serious adverse events (SAE): (1) death; (2)life-threatening; (3)dysfunction/ disability; (4) prolonged hospitalization.

8.6.2 Disposal of cases with SAE

Necessary measures should be taken by researchers to ensure the safety of subjects when SAE occurs.

If SAE has nothing to do with the experimental drug and no serious injuries occur, and patients can be followed up as before, then they can continue; in any other circumstances, patients should quit, it is better to complete a follow-up before quitting.

All SAEs and adverse events that researchers regard probably related to the experimental drug should be monitored, it wasn't until the event was resolved or researchers thought that the event had changed to "chronicity " or "stability" that the follow-up was over.

8.6.3 Records and reports of serious adverse events

Any SAEs occurred during the trail and after withdrawal must be immediately reported to the sponsor and then to the ethics committee within 24 hours. At the same time, researchers should fill the serious adverse event report form (SAE), in which the SAE occurrence time, degree of disease severity, the relationship between the experimental drug and SAE, and the measures taken should be described in detail, and to sign on the report. The contact information of each unit is shown in Table 2.

Table 2 Contact information of SAE

Unit	Contacts	Contact number	E-mail
Shenzhen Kexing Biological Engineering Co., Ltd. of Medicine	Ge Lan	0755-26549161<br>13316899099	Gelan2004@126.com<br>416256042@qq.com

9 Data Management

9.1 Data sourcing and case report form (CRF) filling and transfer

Each subject should complete CRF in duplicate, one retained by the sponsor and the other by the clinical trial units. Without permission, CRF can not be provided to third parties.

After the clinical investigators and monitors reviewed the complete CRFs, they submit them to the statistical units for data entry and management. All procedures require records.

9.2 Data entry and modification

The data administrator of the statistics unit is responsible for data entry and management. All data will be double-entered with EpiData 3.0 software to ensure the accuracy of the data. For problems that exist in CRF, the data administrators will fill the data ready queue (DRO), and inquire the subjects by asking clinical arbitrator for help, they should answer and return it as soon as possible, the data administrators can modify the data according to the answers, and do data confirmation and entry, and they can emit DRO again if necessary.

9.3 Data Verification

Data verification procedures include computerized verification (Edit Check), human checking and data verification meetings. Inconsistent data (Data Query) found in the verification should be promptly corrected or discrepancy reports be issued by the data management, then submit to researchers to make it confirmed, and then make changes.

10 Statistical analysis

10.1 Analysis sets

(1) Full analysis set (FAS): All randomized subjects were included in the groups while in-group cases without any follow-up observations were excluded. The missing data will be replaced by the Last Observation Carry-Forward, so as to evaluate the efficacy and ensure the number of subjects at the initial group is consistent with that of the end.

(2) Per protocol set (PPS): Cases are included in the dataset who meet the trial protocol, measurable main variables, no lack of baseline data, no severe breach of protocol, and evaluatable clinical efficacy.

(3) Statistical analysis of main variables: statistical analysis was done to FAS and PPS, respectively; the demographic, other baseline data, as well as curative effect was analyzed by FAS.

10.2 Statistical method

10.2.1 Descriptive analysis

(1) Normal distribution or not: if the data does not fit the normal distribution, statistical methods will be changed or perform a data conversion;

(2) Outlier: statistics and professional analysis were performed to determine to accept or reject it;

(3) Missing values: the Last Observation Carry-Forward was done when a main therapeutical index of some individuals failed to be observed.

(4) Proportion of drop-out cases: no more than 20%, if exceeded, give a description;

(5) Descriptive statistical analysis: mean, standard deviation, maximum value, minimum value, confidence interval, and so on.

10.2.2 Analysis method

(1) Quantitative data: quantitative data such as age, height, EV71 viral load in stool, fever clearance time, rash healing time, GCS score which meet the normal distribution will take the t test or paired t-test;

(2) Enumeration data: enumeration data such as NICU/RICU incidence rate will take the calibration chi-square test, if the number of cases is less than 10, it will take the Fish's exact test.

10.2.3 Statistics

(1) Reports will be mainly presented with self-explanatory forms, including titles, notes and the number of cases.

(2) Two-sided tests were performed except for non-inferiority test, P value≤0.05 is considered statistically significant.

10.3 Statistical Software

(1) EpiData 3.0, double-entered.

(2) SPSS 15.2.

10.4 Statistic analysis

The statistical analysis plan will be formulated by the principal of statistical analysis and investigators according to the trial protocol, including data management, statistical methods and contents, and to confirm and refine the plan before a formal statistical analysis. Contents are mainly as follows:

(1) Case distribution: to compare the total drop-out rate and the drop-out rate caused by adverse events by Chi-square test.

(2) Comparability analysis: to compare the demographic data and other underlying indicators to test the comparability of the two groups.

(3) Compliance analysis: to record the intake amount and the intake time of experimental drugs of two groups, and to record whether they take banned drugs and food or not.

(4) Validity analysis: the primary variables and the global indicators will be analyzed by PPS and FAS, respectively, the center effect on the curative effect should be taken into consideration since this study is a multi-center trial. For global curative effect indicators, the superiority test will be performed to compare the experimental drug and active control.

(5) Safety analysis: on requirement of the adverse events, tables and figures are used to interpret the adverse events and reactions of both groups (including the number of adverse events, the number and mutation rate of "normal ones transfer to abnormal ones" before and after the experiment), and then chi-square test is used to compare the rates of adverse reactions.

11. Ethics Statement

11.1 Clinical trial plan complied with ethics requirements

(1) The clinical trial protocol is in accordance with the principles of the Declaration of Helsinki.

(2) The clinical trial protocol formulated by principal investigators and the sponsor should be submitted to the Ethics Committee for approval before implementation.

(3) Ethics Committee's opinion could be “agree, agree after making the necessary amendments, disagree, terminate or suspend the approved trial”.

(4)If problems occur during the trial and the protocol needs to be modified, the revised protocol should be implemented upon the Ethics Committee’ approval.

11.2 Privacy protection

Researchers must protect the privacy of the trial subjects. All the documentation of the subjects to the sponsor can only use the identities of the subjects without names and No., researchers must well keep subjects' names and addresses, as well as the in-group forms corresponding to the numbers of subjects. These forms should be well kept by researchers and should not be submitted to the sponsor.

11.3 Informed consent form (ICF)/data protection agreement

It is the researchers’ duty to explain the objective, methods, benefits and potential risks of this trial to the parents of the subjects, and to obtain the written informed consents. By signing the ICF, parents should also allow clinical monitors to have access to the source data to verify the information of the trial, in order to determine the reliability of data.

ICFs must be personally signed and dated by the subjects, and researchers must well keep the ICFs.

12 Protocol modification

When the protocol is approved by the Ethics Committee, if there are significant changes in the trial process, principal investigators should write "modification instruction" and signed personally, and then submit it to the Ethics Committee. If there is no principal changes, it should be discussed and determined by principals of the trial, statisticians, as well as sponsors, and then to inform the other participating units.

13 Case report form(CRF)

Researchers must fill the CRFs completely and accurately. Each CRF represents a clinical subject. No altering can be done to the wrong data or text, just underline it, and then to refill the correct data or text besides, and sign the researcher's name and make date modified.

14 Source record verification

Researchers must properly handle all the data obtained in the course of the trial, in order to ensure the rights and privacy of patients participating in the trial. monitors/auditors/inspectors must be allowed to inspect and check the source information to verify the accuracy and obtain the progress of the study. If they fail to verify the source data, researchers should allow them to further confirm the data quality.

15 Trial summary

After the statistical analysis report is finished by statisticians, the participating units then submit the center summary, and the clinical unit is responsible for the summary report.

16 Responsibilities of participants and rules of article publishing

(1) Sponsors and researchers shall undertake the corresponding responsibilities in accordance with the Good Clinical Practice and principles of this protocol.

(2) Rules of article publishing: after the trials, clinical trial units have the right to publish this clinical study report in article form, and research sponsors own the naming rights.

17 Quality control and management

17.1 Measures to improve the subjects' compliance

(1) In order to ensure the subjects' compliance and a good efficacy, researchers should make the subjects fully understood the significance of the trial, the importance of taking medication as well as schedule review on time through written descriptions and oral explanations. Researchers should guide the subjects properly and inform them of follow-up on time according to the protocol requirements.

(2) Monitor the subjects' compliance by drug counts, compliance = (actual dosage/requested dosage) ×100%. Compliance less than 100% or more than 100% is regarded as significant violations of the trial protocol, that is, patients take 3 consecutive days of the drug can be regarded as a good compliance.

17.2 Quality control and quality assurance systems

Sponsors and researchers should perform their corresponding duties and strict

compliance with the clinical trial protocol, respectively; to develop and follow the Standard Operation Procedure (SOP), as well as ensuring all relevant personnel are in close collaboration and strictly in accordance with GCP and SOP requirements such as audits, inspections, monitoring, to guarantee all information reliable, ensuring that all actions recorded are available.

Researchers should have the deputy of associate chief physicia or beyond and relatively fix.

Determination of diagnosis and treatment evaluation of subjects should be completed by at least one physician or above, and the physician should be relatively fixed.

Researchers should be in accordance with the of CRF requirements, the records of first diagnosis and post-treatment should be itemized according to the fact. Principal investigators should regularly check after the trial is completed and sign the final verification of CRFs.

The unit should keep in touch with the trail center, timely communication, notification and discussion should be done to the problems that are found or exist in early period of the trial, if modification needed, a meeting should be held by the sponsor to discuss the trial scheme and then modify it, the modified trial plan must be resubmitted to the Ethics Committee for review and approval.

Effects of Recombinant Human Interferon α1b on Critically ill Hand, Foot, and Mouth Disease cases associated with Enterovirus 71 Infection: A Multi-center, Randomized, Controlled Clinical Trial with Blank Control

HFMD cases sample registration form

Recruited sequence NO.	Specimen	Name	Gender	Date of Birth	Date of onset	Sampling date	Address and telephone	Clinical diagnosis

Notes: Specimen type can be stool specimen, throat swab specimen, anal swab specimen, or sera sample.

Sampling unit:____________________Sample collector: ____________________ Tel: ____________________

Methods and Requirements of Specimen Collection

(1) Stool specimens: stool samples of the clinical diagnosed HFMD patients are collected during hospitalization with the amount of 5 ~ 8g each stool dated, and transferred into a sterile seal tube immediately after collection, 4 ℃ of temporary storage (within 12h) or below -20 ℃ of cryogenic preservation, biological safety box with ice packs or ice floe is used to transport them to the laboratory. Wherein, the first stool specimen after admission and the one prior to discharge of hospital are used to do the viral load testing.

(2) Anal swab: If the stool samples can not be collected, anal swab specimens can be collected instead, but it can not be used to do quantitive analysis of EV71 virus load. Anal swab sampling method: a cotton swab dampened with normal saline is moderately inserted into the patient's anus, and then rotated in the same direction, take out the cotton swab into a sampling tube with 3 ~ 5ml preservation solution(to maintain liquid, or saline solution is recommended to maintain), and then break it near the stick top, close and fasten the lid and seal. Storage and transportation are the same to that of stool specimens.

(3) Throat swabs: the throat swabs are collected on the first day and then every other day between admission and inhalation of recombinant human interferon α-1b, it can be used to observe the virus duration in subsequent research. Swab sampling methods: special sampling cotton swab is used to moderately wipe the postpharyngeal gland and tonsils of both sides, avoid touching the tongue, the cotton swab is put into the sampling tube containing 3 ~ 5ml preservation solution (maintained or saline) as quickly as possible, and then break it near the stick top, close and fasten the lid and seal. Storage and transportation are the same to that of stool specimens.

(4) Serum samples: double-paired serum samples of the acute phase (onset time:0~5d) and recovery (onset time: 14 ~ 30d) are collected for antibody detection. When IgM detected, blood (onset time7 ~ 20d) is required. 3 ~ 5ml vein blood is collected, place it in a vacuum sterile blood tube, after the coagulation, blood serum is obtained by centrifugation and then transferred into a sterile tube with washers and tighten the lid

and seal. In order not to increase the difficulty of experimental observations, clinical serum specimens posterior to the blood testing can be collected as the follow-up specimens. Storage and transportation are the same to that of stool specimens.

(5) Cerebrospinal fluid (CSF) specimens: If CSF specimens of cases complicated with neurological symptoms is collected for clinical laboratory testing, then it will be collected by our task force to do subsequent research. Storage and transportation are the same to that of stool specimens.
