# Supplementary material for: Clinical Efficacy of Therapy with Recombinant Human Interferon α1b in Hand, Foot, and Mouth Disease with Enterovirus 71 Infection
Source: PLoS One. 2016 Feb 16;11(2):e0148907. doi: 10.1371/journal.pone.0148907 (PMC4755579; doi:10.1371/journal.pone.0148907)
Supplement: S1 Table — (PDF) [file pone.0148907.s004.pdf]

S1 Table 1. The respiratory rates and heart rates of the HFMD patients every day during clinical trials

Groups	first day	second day	third day	fourth day	fifth day
**Respiratory rates (times/min)**					
Control group (n=97)	32.93±3.17	31.83±6.88	30.09±6.08	27.25±4.35	26.43±4.59
Ultrasonic aerosol inhalation group (n=102)	32.10±6.62	30.35±6.24	28.84±6.54	26.53±4.70	25.40±4.28
Intramuscular injection group (n=75)	32.38±6.67	35.25±7.56	31.29±6.27	28.77±6.15	27.68±4.79
F value (*P* value)	0.258(0.772)	10.052(0.000)	2.867(0.59)	3.757(0.25)	4.601(0.11)
**Heart rates (times/min)**					
Control group (n=97)	130.40±11.47	152.82±23.19	126.53±11.73	121.01±8.42	118.18±8.04
Ultrasonic aerosol inhalation group (n=102)	131.55±12.43	128.64±12.24	123.66±9.88	118.97±8.29	130.77±15.38
Intramuscular injection group (n=75)	128.87±6.97	139.61±11.75	131.36±10.31	141.23±31.04	123.07±8.29
F value (*P* value)	1.392(0.250)	0.836(0.435)	10.206(0.001)	1.9972(0.142)	0.638(0.529)
